# Dietary Fiber Supplementation Reduces Systolic Blood Pressure in High-Risk Patients with Type 2 Diabetes and Hypertension: A Secondary Analysis of a Randomized Trial

**DOI:** 10.3390/nu18182945

**Published:** 2026-09-08

**Authors:** José Luis Pino-Villalón, Bastian Parada-Flores, Miguel Arredondo, Roberto Fuentealba, Jessica Noack, Exal Garcia-Carrillo

**Affiliations:** 1 Escuela de Nutrición y Dietética, Facultad de Salud, Universidad Santo Tomás, Talca 3460000, Chile; jpino9@santotomas.cl (J.L.P.-V.); paradafloresbastian@gmail.com (B.P.-F.); 2 Food Observatory (ODA360), Health Prevention and Care Research Center (+SALUD), Faculty of Health Sciences, Universidad Santo Tomás, Santiago 8370003, Chile; 3 Department of Physical Activity Sciences, Faculty of Education Sciences, Universidad Católica del Maule, Talca 3480112, Chile; 4 Micronutrient Laboratory, Institute of Nutrition and Food Technology (INTA), Universidad de Chile, Santiago 7830490, Chile; miguel.arredondo@inta.uchile.cl; 5 Escuela de Enfermería, Facultad de Salud, Universidad Santo Tomás, Talca 3460000, Chile; rfuentealba5@santotomas.cl (R.F.); jnoack@santotomas.cl (J.N.); 6 Department of Physical Activity Sciences, Universidad de Los Lagos, Osorno 5290000, Chile

**Keywords:** dietary fiber, hypertension, diabetes mellitus, type 2, gut microbiota, inflammation, beta-glucans, cellulose, gastrointestinal microbiome, biomarkers

## Abstract

**Background/Objectives**: Dietary fiber supplementation has been associated with blood pressure (BP) reduction, but evidence comparing soluble (SDF) and insoluble (IDF) fiber types in high-risk populations is limited. This study aimed to compare the effects of SDF and IDF supplementation on BP and to explore correlations between changes in BP and alterations in gut microbiota composition and serum biomarkers in adults with type 2 diabetes (T2D) and hypertension. **Methods**: This secondary analysis was based on a 12-week, randomized, double-blind, parallel-group clinical trial. Thirty-five adults with T2D and hypertension were assigned to receive either 5 g/day of oat β-glucan (SDF, n = 19) or microcrystalline cellulose (IDF, n = 16). BP, gut microbiota composition (quantitative polymerase chain reaction; qPCR), and inflammatory biomarkers were assessed at baseline and after the intervention. **Results**: The mean age of the participants was 50.7 ± 5.7 years, and 77% were female. Both groups exhibited comparable reductions in systolic blood pressure (SBP) (SDF: −11.2 ± 20.9 mmHg; IDF: −11.6 ± 17.0 mmHg; *p* = 0.965 between groups). In the SDF group, significant positive correlations were observed between increases in *Firmicutes* abundance and changes in diastolic blood pressure (DBP) (*r* = 0.90, *p* < 0.01), as well as between increases in *Bifidobacterium* spp. abundance and changes in DBP (*r* = 0.90, *p* < 0.05). **Conclusions**: Supplementation with either soluble or IDF was associated with reductions in SBP in this sample of adults with T2D and hypertension. The observed associations between changes in gut microbiota composition and BP should be considered exploratory and interpreted with caution. Placebo-controlled studies are required to confirm these findings and elucidate the underlying biological mechanisms.

## 1. Introduction

Hypertension is a major risk factor for cardiovascular morbidity and mortality worldwide [1]. When hypertension coexists with type 2 diabetes (T2D), the risk of major cardiovascular events, including myocardial infarction, stroke, and heart failure, increases synergistically, exceeding the additive risk associated with either condition alone [2]. Managing this condition in high-risk populations, such as individuals with T2D, requires multidimensional strategies in which nutrition plays a central role. In this context, dietary fiber, a nondigestible carbohydrate, has emerged as an important dietary component for promoting cardiometabolic health [3]. However, globally, dietary fiber intake remains well below the recommended levels (25–35 g/day), and this gap is particularly concerning in populations with metabolic diseases, where mean intake rarely exceeds 15–18 g/day [4]. This dietary deficiency represents a missed opportunity to modulate blood pressure (BP) and other residual cardiovascular risk factors.

Current evidence on the relationship between dietary fiber and BP is derived primarily from meta-analyses of clinical trials, which have reported modest but significant reductions in systolic blood pressure (SBP) following supplementation with viscous soluble fibers, such as oat β-glucan and psyllium. These effects appear to be particularly pronounced in hypertensive populations, with average reductions ranging from −1.4 to −2.9 mmHg [5,6]. However, these meta-analyses highlight important limitations: most studies are of short duration (≤8 weeks), include heterogeneous populations ranging from healthy individuals to patients with hypertension, and, crucially, do not directly compare different types of dietary fiber within the same experimental design [5].

In contrast, evidence regarding the direct effects of insoluble fiber on BP is derived primarily from observational studies, such as the INTERMAP study, which reported inverse associations between insoluble fiber intake (IFI) and BP [7], as well as from prospective studies linking greater diversity of insoluble fiber sources with a lower risk of incident hypertension (relative risk [RR]~0.50) [8]. However, in the absence of controlled trials directly comparing both types of fiber within the same high-risk population, uncertainty remains as to whether insoluble fiber exerts a true antihypertensive effect and whether its magnitude is comparable to that of soluble fiber.

This uncertainty provides a strong rationale for the selection of these two types of fiber in the present study. Oat β-glucan (soluble fiber) was selected because it has the strongest experimental evidence supporting its metabolic and cardiovascular benefits [9,10]. In contrast, microcrystalline cellulose (insoluble fiber) was selected not as an “inert placebo,” as commonly used in the literature [11], but rather as an active comparator to test the hypothesis that increasing total dietary fiber (TDF) intake, regardless of solubility, may provide comparable benefits. This comparison is clinically relevant because, if both types of fiber prove effective, it could expand dietary options for patients who poorly tolerate viscous fibers (e.g., due to abdominal bloating) or who have specific dietary preferences, thereby facilitating long-term adherence. To date, no randomized clinical trial has specifically compared β-glucan with microcrystalline cellulose in patients with concomitant T2D and hypertension. Therefore, this secondary exploratory study addresses a relevant evidence gap.

Several mechanisms have been proposed to explain the effects of dietary fiber on BP, and these mechanisms may differ according to fiber type. Soluble fibers, such as β-glucan, form viscous gels within the gastrointestinal tract and are extensively fermented by the gut microbiota, generating SCFAs, including acetate, propionate, and butyrate [12]. These SCFAs activate receptors such as GPR41, GPR43, and Olfr78 in the intestinal epithelium and endothelial cells, thereby modulating renin secretion and peripheral vascular tone [13,14]. In contrast, insoluble fiber, such as cellulose, has traditionally been considered poorly fermentable; however, recent evidence suggests that it can modify gut microbiota composition and increase butyrate production in ex vivo and in vivo models [15,16]. In addition, insoluble fiber may improve intestinal barrier integrity, thereby reducing lipopolysaccharide translocation and subsequent low-grade systemic inflammation [17], while also increasing fecal sodium excretion [18]. Thus, both types of fiber may converge on pathways involved in cardiovascular regulation, including the modulation of inflammation, hormonal regulation, and fluid and electrolyte balance.

Through the production of microbial metabolites and the modulation of inflammatory pathways, the gut microbiota has been proposed as a key regulator of BP [19]. Individuals with hypertension commonly exhibit gut dysbiosis characterized by reduced microbial diversity and alterations in the *Firmicutes*/*Bacteroidetes* ratio [20]. These microbial alterations have been associated with systemic disturbances, including low-grade inflammation, hormonal resistance, and metabolic dysfunction, all of which represent key pathophysiological mechanisms underlying hypertension. Furthermore, biomarkers such as cytokines (TNF-α and IL-6), cortisol, leptin, resistin, glucagon-like peptide-1 (GLP-1), peptide YY (PYY), and zonulin have been implicated in BP regulation [21,22,23]. However, no study to date has comprehensively assessed the impact of soluble versus insoluble fiber supplementation on BP, the abundance of key bacterial taxa, and a broad panel of biochemical mediators within the same cohort of patients with T2D and hypertension. This lack of integration limits our understanding of which biological pathways, including inflammatory, hormonal, and intestinal barrier pathways, predominantly mediate the effects of each type of fiber on BP.

Therefore, the aim of this secondary analysis was to explore the associations between the effects of 12 weeks of SDF (oat β-glucan) and IDF (microcrystalline cellulose) supplementation on SBP and DBP in adults with T2D and hypertension. In addition, we explored correlations between intervention-induced changes in the relative abundance of gut bacterial taxa and changes in BP, as well as association with an extensive panel of serum biomarkers related to inflammation, hormonal regulation, and intestinal permeability. We hypothesized that both types of fiber would reduce SBP to a similar extent, and that these reductions would be differentially associated with changes in the gut microbiota and specific metabolic and inflammatory pathways, thereby providing insights to inform future studies.

## 2. Materials and Methods

### 2.1. Study Design

This study is a secondary analysis of a previously published randomized, double-blind, parallel-group clinical trial [16]. The primary objective of the original trial was to evaluate the effects of oat β-glucan supplementation on glycemic control and metabolic parameters in individuals with T2D. Because the recruited cohort had a high prevalence of hypertension (93% of participants), the present secondary analysis was conducted to specifically investigate the effects of dietary fiber supplementation on BP in this hypertensive subgroup. Accordingly, this analysis should be considered exploratory.

A total of 44 participants with T2D were randomly assigned to one of two intervention groups. The randomization sequence was generated using a computerized procedure with variable block sizes [4,5,6] and a 1:1 allocation ratio to ensure numerical balance between groups throughout the recruitment period. One group received 5 g/day of oat β-glucan (soluble dietary fiber [SDF]), while the other group received 5 g/day of microcrystalline cellulose (insoluble dietary fiber [IDF]). No additional placebo group was included because the original study design compared two active intervention arms. The allocation codes were not disclosed until completion of the data analysis. The study protocol was approved by the Scientific Ethics Committee of the Maule Health Service, Chile, and was registered at ClinicalTrials.gov (Identifier: NCT04299763).

### 2.2. Participants

Participants were recruited from a Family Health Center (FHC) in Talca, Chile. The inclusion and exclusion criteria of the original study [16] were as follows.

Inclusion criteria: individuals with a diagnosis of T2D, receiving oral glucose-lowering treatment with metformin, aged between 30 and 60 years, with a disease duration of more than 1 year and less than 10 years, without major chronic complications, glycated hemoglobin (HbA1c) levels between 7% and 9%, of either sex, and a body mass index (BMI) > 30 kg/m^2^.

Exclusion criteria: pregnant women; individuals with a history of acute or chronic intestinal diseases; use of medications that could interfere with the gut microbiota, including antibiotics, anti-inflammatory drugs, laxatives, or prokinetic agents; current smoking; changes in usual diabetes treatment during the study, including initiation or dose escalation of metformin, insulin, or other antidiabetic medications; regular consumption of specific probiotics or prebiotics during the 2 months before enrollment; and treatment with DPP-4 inhibitors or α-amylase inhibitors.

**Figure 1:**
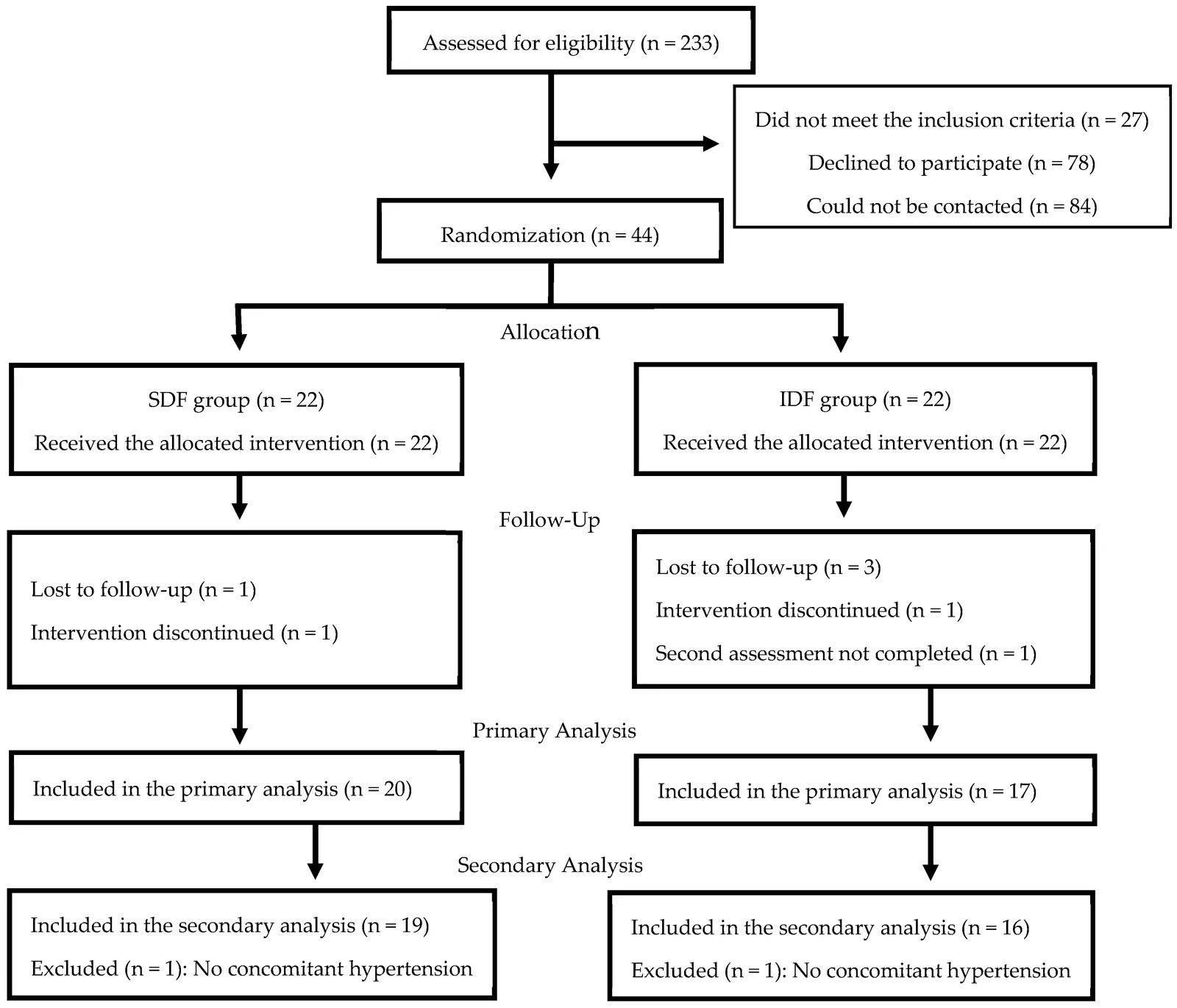
Participant flow and study analyses.

Study population analyzed in the present study: Of the 44 participants initially randomized, 37 completed the trial (17 in the IDF group and 20 in the SDF group). For the present secondary analysis, only participants with concomitant hypertension were included, resulting in a final sample of 35 participants (16 in the IDF group and 19 in the SDF group) (Figure 1).

The sample size was originally calculated based on the primary outcome of the parent study, namely the effect of β-glucan supplementation on HbA1c. A sample of 22 participants per group was required to detect a difference of 0.4 percentage points in HbA1c, with 80% power and a two-sided significance level of 0.05, assuming a 20% attrition rate. For the present secondary analysis focusing on BP in the hypertensive subpopulation, a post hoc power calculation indicated 82% power to detect a clinically relevant between-group difference of 20 mmHg in SBP, assuming a pooled standard deviation of 18 mmHg and a two-sided α level of 0.05. Therefore, the exploratory nature and limited statistical power of the subgroup analyses, particularly those examining correlations with gut microbiota composition and serum biomarkers, which were not considered in the original sample size calculation, should be clearly acknowledged. Accordingly, the findings from these analyses should be interpreted as hypothesis-generating and require confirmation in studies specifically designed and adequately powered to address these research questions.

The sample size was calculated based on the primary objective of the original study, namely, the effect of β-glucan supplementation on HbA1c. This calculation required 22 participants per group to detect a 0.4 percentage-point difference in HbA1c, with 80% power and a significance level of 0.05, assuming a 20% attrition rate. For the present secondary analysis, which focused on BP in the hypertensive subpopulation, a post hoc power analysis indicated 82% power to detect a clinically relevant between-group difference of 20 mmHg in SBP (pooled standard deviation: 18 mmHg; two-sided α = 0.05).

Therefore, the exploratory nature of this secondary analysis and the limited statistical power of the subgroup analyses, particularly those examining correlations with gut microbiota and serum biomarkers, which were not considered in the original sample size calculation, should be clearly acknowledged. Accordingly, the findings from these analyses should be interpreted as hypothesis-generating and require confirmation in studies specifically designed to address these research questions.

### 2.3. Intervention

Participants were assigned to one of two clinically comparable groups, each comprising adults with T2D and hypertension. Over a 12-week period, one group received SDF supplementation, whereas the other received IDF. A 2-week run-in period preceded the intervention for all participants.

The SDF group received purified oat β-glucan powder (98.1% purity; Xi’an Yaochang Co., Ltd., Xi’an, China), whereas the IDF group received microcrystalline cellulose (98% purity; Avicel PH101, FMC BioPolymer, Philadelphia, PA, USA). Both supplements were provided in containers equipped with an integrated measuring scoop. Twenty-four-hour dietary recalls were collected throughout the study to assess dietary fiber intake from foods consumed outside the intervention. Twenty-four-hour dietary recalls (24-h DRs) were conducted at baseline and at the end of the intervention to assess dietary fiber intake from sources outside the study intervention.

The trial was conducted under double-blind conditions. Participants, intervention-administration personnel, healthcare personnel, and laboratory analysts were unaware of group allocation. The supplements (IDF and SDF) were provided as powders with similar appearance and texture and were packaged in identical coded containers. Although the two compounds may differ in their physical properties (e.g., bulk density and behavior in solution), participants were instructed to consume the powder mixed with liquids or semi-solid foods (milk, yogurt, or fruit juice) at room temperature, thereby minimizing the perception of organoleptic differences. At the end of the study, a supplement perception questionnaire assessing odor, taste, consistency, and appearance was administered to evaluate the success of blinding. No significant between-group differences were observed in the ratings.

Adherence to the daily supplement intake (5 g/day) was assessed using two complementary strategies. First, the containers returned at the end of each supplementation period (every 4 weeks) were physically counted, and adherence was calculated as the proportion of consumed doses relative to the total number of expected doses. Second, 24-h DRs were administered at baseline and at the end of the intervention to identify potential additional sources of dietary fiber outside the study intervention. Weekly telephone calls were also conducted to monitor adherence and record any gastrointestinal symptoms. Mean adherence was 93.2% in the SDF group and 91.8% in the IDF group, with no significant between-group difference (*p* = 0.412, Student’s t-test). No participant reported consuming other fiber supplements or probiotics during the study period.

### 2.4. Blood Pressure Assessment

Systolic and diastolic BP were assessed at two time points: at baseline (week 0) and following the 12-week intervention (week 12). All measurements were conducted by the same trained investigator, who was previously certified in standardized BP measurement techniques in accordance with World Health Organization (WHO) guidelines, thereby minimizing interobserver variability.

Assessments were conducted at participants’ homes during morning visits (between 7:00 and 10:00 a.m.). To ensure comparable conditions between baseline and follow-up assessments, each measurement was scheduled within ±1 h of the participant’s baseline assessment time. In addition, participants completed an overnight fast of 8–12 h before each visit, which was confirmed by telephone beforehand.

BP was measured using a calibrated digital sphygmomanometer (Omron HEM-CR24, Omron, Bannockburn, IL, USA). Three consecutive measurements of SBP and DBP were obtained at intervals of at least 2 min, and the average of the three readings was used for statistical analyses.

The use of antihypertensive medication was verified in all participants included in this analysis. All participants were receiving stable treatment with either enalapril or losartan, both provided through the Chilean public healthcare system. The distribution of antihypertensive medications was comparable between the SDF and IDF groups (χ^2^ = 0.80, df = 1, *p* = 0.370). Although these medications have been reported to influence gut microbiota composition [24], their balanced distribution between groups minimizes their potential confounding effect, allowing the observed between-group differences to be reasonably attributed to dietary fiber intervention.

### 2.5. Gut Microbiota Analysis

A detailed description of the gut microbiota analysis has been published previously [16]. Fecal samples were collected from participants at baseline and at the end of the intervention. Genomic DNA was extracted using a commercial DNA extraction kit.

The relative abundance of selected bacterial taxa was quantified by quantitative real-time polymerase chain reaction (qPCR) as previously described [16]. Previously validated primer sets were used to quantify total bacteria; the phyla *Firmicutes*, *Bacteroidetes*, and *Verrucomicrobia*; and the bacterial groups *Lactobacillus* spp., *Bifidobacterium* spp., *Akkermansia muciniphila*, and butyrate-producing bacteria. Relative quantification was performed using the 2−ΔΔCt method based on quantification cycle (Cq) values.

### 2.6. Biochemical Marker Assessment

Fasting blood samples were collected at baseline and at the end of the intervention to quantify inflammatory, hormonal, and metabolic biomarkers. Serum concentrations of tumor necrosis factor-α (TNF-α), interleukins (IL-6, IL-1β, IL-8, and IL-10), cortisol, leptin, resistin, PYY, ghrelin, C-peptide, and GLP-1 were determined using the Magnetic Luminex^®^ assay (R&D Systems Inc., Minneapolis, MN, USA) and analyzed with a MAGPIX^®^ system (Luminex Corporation, Austin, TX, USA). Two assay panels were used according to the manufacturers’ instructions: the Human Magnetic Luminex Screening Assay (R&D Systems, Minneapolis, MN, USA) for interleukins and the Metabolism/Obesity Panel (Thermo Fisher Scientific, Waltham, MA, USA) for hormonal biomarkers.

### 2.7. Statistical Analysis

BP data are presented as mean ± standard deviation (SD). Within-group differences were assessed using paired *t*-tests for normally distributed variables or the Wilcoxon signed-rank test for non-normally distributed variables, as determined by the Shapiro–Wilk test.

Two-way mixed repeated-measures analysis of variance (ANOVA) was performed to evaluate the effects of group (SDF vs. IDF), time (baseline vs. week 12), and the group × time interaction on systolic and diastolic BP. Time was treated as the within-subject factor and group as the between-subject factor.

Given the exploratory nature of this secondary analysis and the large number of correlations examined (changes in 8 bacterial taxa × 19 serum biomarkers × 2 BP variables = 304 tests per group), no adjustment for multiple comparisons was applied. This decision was based on the concern that such adjustment would substantially increase the risk of Type II error in a study with a limited sample size, particularly given that the objective was not to test causal hypotheses but rather to identify exploratory signals that may inform future research. Nevertheless, we explicitly acknowledge that conducting multiple statistical tests at a significance threshold of *p* < 0.05 increases the risk of Type I error. Therefore, all statistically significant correlation findings were interpreted with considerable caution as hypothesis-generating rather than confirmatory evidence. Exact *p*-values and 95% confidence intervals (95% CIs) were reported for statistically significant correlation coefficients. Statistical analyses were performed using IBM SPSS Statistics, version 30 (IBM Corp., Armonk, NY, USA).

## 3. Results

Of the 44 participants initially randomized in the original trial, 7 did not complete the 12-week follow-up (5 in the IDF group and 2 in the SDF group). All withdrawals were due to personal reasons unrelated to the intervention, adverse events, or supplement intolerance. Thus, 37 participants completed the trial (17 in the IDF group and 20 in the SDF group). Because the present analysis specifically focused on participants with concomitant hypertension, 2 participants (1 from each group) were excluded because they did not meet the diagnostic criteria for hypertension at baseline. The final sample for the secondary analysis comprised 35 participants: 16 in the IDF group and 19 in the SDF group. Figure 1 presents the CONSORT flow diagram detailing participant losses and exclusions.

The groups were comparable at baseline, except for fasting blood glucose, which was higher in the IDF group. The specific baseline characteristics of each group are presented in Table 1.

Energy and nutrient intake, including macronutrients and dietary fiber, was assessed using 24-h dietary recalls at baseline and at the end of the intervention. No significant differences were observed in dietary fiber intake from usual food sources (*p* > 0.05), excluding the 5 g/day provided by the study supplement (Table 2).

Therefore, total fiber intake (dietary intake plus supplementation) was approximately 27 g/day in both groups, approaching international recommendations of 25–38 g/day.

The absence of changes in dietary intake throughout the study suggests that the observed changes in BP and metabolic parameters were reasonably attributable to the dietary fiber supplementation rather than to spontaneous modifications in participants’ usual dietary intake.

Table 3 presents the absolute BP values at baseline and at the end of the intervention, as well as the changes observed in each group. Both groups showed clinically relevant reductions in SBP: −11.2 ± 20.9 mmHg in the SDF group and −11.6 ± 17.0 mmHg in the IDF group. A two-way repeated-measures ANOVA revealed a significant main effect of time on SBP (F(1,34) = 6.340; *p* = 0.020), with an overall reduction of −11.4 mmHg (95% CI: −16.8 to −6.0). No significant between-group effect was observed (group effect: F(1,34) = 1.132; *p* = 0.299), nor was there a significant group × time interaction (F(1,34) = 0.018; *p* = 0.893).

For DBP, no significant overall changes were observed (time effect: F(1,34) = 0.047; *p* = 0.830; overall change: −0.95 mmHg; 95% CI: −4.04 to 2.14), and neither the group effect nor the group × time interaction was statistically significant.

Table 4 presents the correlation coefficients between changes in bacterial populations and changes in BP according to the type of fiber consumed. In the IDF group, strong but non-significant negative correlations were observed between changes in total bacteria and SBP (r = −0.67, *p* = 0.102), and between changes in Akkermansia muciniphila and SBP (r = −0.67, *p* = 0.253). In the SDF group, significant positive correlations were observed between changes in Firmicutes and DBP (r = 0.90, 95% CI: 0.74–0.96; *p* = 0.006), and between changes in Bifidobacterium spp. and DBP (r = 0.90, 95% CI: 0.62–0.98; *p* = 0.037). No other statistically significant correlations were observed.

No statistically significant correlations were observed between changes in serum biomarkers (TNF-α, IL-6, IL-1β, IL-8, IL-10, cortisol, FGF-23, leptin, resistin, peptide YY, ghrelin, C-peptide, GLP-1, and zonulin) and changes in BP in either group (Table 5).

## 4. Discussion

In this secondary analysis of a randomized clinical trial, 12-week supplementation with either SDF (oat β-glucan) or IDF (microcrystalline cellulose) was associated with significant and clinically meaningful reductions in SBP in patients with T2D and hypertension, with no significant differences between fiber types (overall reduction: −11.4 mmHg; 95% CI: −16.8 to −6.0 mmHg; *p* = 0.020). In contrast, no significant changes were observed in DBP. However, it is crucial to emphasize that, because the study did not include a placebo or non-intervention control group, the observed antihypertensive effect cannot be specifically attributed to dietary fiber supplementation. Therefore, these findings should be considered exclusively hypothesis-generating and exploratory.

The magnitude of SBP reduction observed in the present study is consistent with previous meta-analyses reporting antihypertensive effects of soluble dietary fiber, particularly in hypertensive populations [5]. Specifically, oat β-glucan has been shown to produce modest reductions in SBP in adults with overweight or obesity (−1.38 mmHg; 95% CI: −2.66 to −0.09 mmHg; *p* = 0.036) when administered at doses ≥3 g/day for at least six weeks [25]. Conversely, other studies conducted in individuals with mild hypertension (approximately 130/80 mmHg) using cookies enriched with 4 g of oat β-glucan for four weeks found no significant effects on either SBP or DBP [9]. Nevertheless, the study by Evans et al. [26] is particularly noteworthy, as it demonstrated that β-glucan exerted the greatest BP-lowering effect among all dietary fibers evaluated in healthy individuals, reducing SBP by 2.9 mmHg, compared with an overall reduction of only 0.9 mmHg for dietary fiber as a whole [26]. Furthermore, the antihypertensive effect of β-glucan appears to be more pronounced in hypertensive individuals with obesity (BMI ≥ 31.5 kg/m^2^), with reported reductions of 8.3 mmHg in SBP (*p* = 0.008) and 3.9 mmHg in DBP (*p* = 0.018) [27]. This finding is particularly relevant because participants in the present study had a mean BMI of 34.2 ± 7.04 kg/m^2^ in the IDF group and 33.2 ± 5.16 kg/m^2^ in the SDF group [16]. Therefore, the antihypertensive effects of β-glucan may be particularly relevant in individuals with concomitant obesity and hypertension.

The most noteworthy finding of this secondary analysis, however, is that IDF (microcrystalline cellulose) produced an antihypertensive effect comparable in magnitude to that of SDF. This observation challenges the conventional paradigm that attributes superior BP-lowering effects to viscous and fermentable fibers [6]. Although direct evidence regarding IDF is largely derived from observational studies such as INTERMAP [7], prospective studies have also reported that greater diversity in insoluble fiber intake is associated with a lower risk of incident hypertension (HR = 0.50; 95% CI: 0.45–0.55) [8]. Direct evidence supporting an antihypertensive effect of microcrystalline cellulose (MCC) remains scarce. Indeed, in the biomedical literature, MCC is widely used as an inert placebo or control in clinical trials [11], including studies evaluating interventions targeting BP [28], based on the assumption that it lacks relevant pharmacological or metabolic activity. However, the present findings raise reasonable questions regarding this assumption. In hypertensive older adults with normal nutritional status (BMI 21.6 ± 0.7 kg/m^2^), supplementation with 600 mg/day of MCC for seven days did not reduce BP [29]. Nevertheless, experimental studies in animal models have shown that MCC may reduce food intake and body weight, improve lipid metabolism, and increase SCFAs production [30], although its direct effects on BP have not been thoroughly investigated. Collectively, our findings suggest that increasing TDF intake, regardless of its solubility (β-glucan or MCC), may represent an equally effective dietary strategy for BP management in individuals with T2D.

The comparable effects observed for soluble and IDF are biologically plausible despite their distinct mechanisms of action, as both pathways may ultimately converge to modulate BP. Soluble fiber (β-glucan) is traditionally thought to exert its effects through microbial fermentation, leading to the production of SCFAs, primarily butyrate, propionate, and acetate, which activate systemic receptors including GPR41, GPR43, Olfr78, GPR109A, and HDAC9. These signaling pathways regulate renin secretion, vascular tone, renal sodium handling, and endothelial function [13,14,31]. In contrast, insoluble fiber (cellulose) may primarily act by increasing fecal sodium excretion [10] and improving intestinal barrier integrity, thereby reducing metabolic endotoxemia [17]. These mechanisms (i.e., attenuation of systemic inflammation and improved sodium homeostasis) may act synergistically and contribute to the similar BP responses observed. Interestingly, ex vivo studies have shown that microbial metabolism of MCC generates higher butyrate production than a commercial prebiotic/probiotic formulation or no treatment [30]. Moreover, our previous findings demonstrated that MCC supplementation increased the abundance of butyrate-producing bacteria in humans [16], which may partially explain the antihypertensive effects observed in the present study [32], particularly in individuals with T2D [33].

The significant positive correlations between increases in *Firmicutes* and *Bifidobacterium* spp. abundance and changes in DBP observed in the SDF group (*r* = 0.90) were unexpected and challenge the conventional view that these taxa exert beneficial effects on BP regulation. *Bifidobacterium* is generally regarded as a probiotic genus that contributes to BP regulation through SCFAs production [34]. However, in the presence of pre-existing gut dysbiosis, a hallmark of both T2D and hypertension [35,36], rapid fermentation of SDF may promote disproportionate expansion of specific bacterial taxa, thereby altering the overall metabolic profile. Although SCFAs such as butyrate generally exert vasodilatory effects [37], an unbalanced fermentation profile may produce transient or even adverse effects on vascular tone, particularly with respect to diastolic function [38]. Importantly, these statistically significant correlations were derived from a very small sample and therefore should be interpreted with caution, as they do not establish causal relationships or robust differential responses between dietary fiber types.

Several limitations of this study should be acknowledged. First, the relatively small sample size in each group (n = 16 and n = 19) limited the statistical power to detect subtle between-group differences and increased the likelihood of type II error. Second, as this was a secondary analysis, the original trial was not specifically designed to evaluate BP outcomes, and the absence of a placebo group precludes attributing the observed reductions exclusively to dietary fiber supplementation. Third, the 12-week intervention may have been insufficient to detect sustained changes in gut microbiota composition and their long-term effects on BP. Fourth, the present study was based on correlation analyses. Although the original trial was randomized to compare the two types of dietary fiber, the analyses examining associations between changes in gut microbiota and serum biomarkers and changes in BP were exploratory and cannot be interpreted as evidence of causal relationships. A positive or negative correlation does not demonstrate that changes in the gut microbiota cause changes in BP; rather, it indicates that both variables changed concomitantly within this specific sample. Mechanistic studies are required to determine whether these associations reflect causal relationships.

Fifth, the correlation analyses were conducted without adjustment for multiple comparisons, which increases the risk of false-positive findings. Although this approach was chosen to avoid overlooking potential biological signals in an exploratory analysis, statistically significant findings should be interpreted with caution and require replication in independent cohorts. Finally, measurements of fecal SCFAs would have provided a more direct mechanistic link between microbial alterations and BP regulation. Despite these limitations, the study has several important strengths. The randomized, double-blind design with an active comparator (microcrystalline cellulose) ensured a high degree of internal validity. BP measurements were performed using standardized procedures by trained personnel. In addition, the simultaneous assessment of gut microbiota composition and an extensive panel of inflammatory, hormonal, and metabolic biomarkers provided a comprehensive evaluation of potential mechanisms underlying the observed effects. Finally, the inclusion of patients with T2D and hypertension enhances the clinical relevance of the findings, as this population is at particularly high cardiovascular risk.

## 5. Conclusions

In this secondary analysis of a randomized clinical trial, significant and clinically relevant reductions in SBP were observed in patients with T2D and hypertension who consumed 5 g/day of either soluble fiber (oat β-glucan) or insoluble fiber (microcrystalline cellulose) for 12 weeks. The magnitude of the SBP reduction was comparable between the two fiber types, with an overall change of −11.4 mmHg (95% CI: −16.8 to −6.0 mmHg; *p* = 0.020). No significant changes in DBP were observed in either group. The observed associations with the gut microbiota were exploratory and should be interpreted cautiously given the limited sample size and the large number of statistical tests performed, which increase the risk of spurious findings. Further studies with greater statistical power and specifically designed experimental approaches are needed to confirm these findings and elucidate the underlying mechanisms.

Although these findings suggest that increasing TDF intake, regardless of fiber type, may represent an accessible nutritional strategy for the management of hypertension, it should be emphasized that the study design does not allow a specific antihypertensive effect to be attributed to dietary fiber itself. The absence of a placebo group precludes determining whether the observed reduction was specifically attributable to the administered fiber, to nonspecific effects of supplementation, or to other uncontrolled factors, such as unmeasured changes in lifestyle or stabilization of antihypertensive pharmacological treatment during the study period.

The observation that IDF produced a BP reduction comparable to that of SDF represents an exploratory hypothesis that warrants reconsideration of the role of insoluble fiber in BP regulation. Randomized placebo-controlled clinical trials with larger sample sizes and mechanistic assessments, including measurements of SCFAs and fecal sodium excretion, are needed to confirm these findings and to elucidate the underlying physiological mechanisms. Finally, although these findings do not allow for causal inferences, they support the recommendation to increase TDF intake.

## Figures and Tables

**Table 1:** Characteristics of the Hypertensive Subsample with Type 2 Diabetes (n = 35).

Variable	Total	IDF	SDF	*p*-Value
Sex, M/F	8/27	3/13	5/14	
Age (years)	50.9 ± 5.6	52.8 ± 3.6	49.7 ± 6.7	0.116
BMI (kg/m^2^)	34.6 ± 5.4	35.3 ± 6.4	34.0 ± 4.45	0.601
Body weight (kg)	86.1 ± 14.3	82.5 ± 14.9	89.1 ± 13.5	0.180
Duration of T2D (years)	7.26 ± 5.12	6.94 ± 4.0	7.53 ± 6.0	0.732
HbA1c (%)	8.87 ± 1.61	8.69 ± 1.74	9.02 ± 1.53	0.550
Fasting blood glucose (mg/dL)	193 ± 58	218 ± 61.8	173 ± 46.3	0.019
Total cholesterol (mg/dL)	148 ± 56.2	148 ± 64.8	148 ± 49.8	0.995
Triglycerides (mg/dL)	261 ± 137	260.8 ± 154	273 ± 132	0.801
SBP (mmHg)	143 ± 20.0	148 ± 17.3	147 ± 19.1	0.760
DBP (mmHg)	80.5 ± 12.3	82.7 ± 11.2	83.8 ± 13.8	0.887

**Table 2:** Dietary intake at baseline and at the end of the intervention according to treatment group.

Variable	IDF Baseline (n = 16)	IDF Week 12 (n = 16)	IDF Change (Δ)	SDF Baseline (n = 19)	SDF Week 12 (n = 19)	SDF Change (Δ)	*p*-Value Baseline *	*p*-Value Week 12 ‡	*p*-Value Δ †
Energy (kcal/day)	1868 ± 269	1716 ± 397	−153 ± 338	2028 ± 417	2050 ± 579	−21.3 ± 662	0.182	0.139	0.471
Protein (g/day)	75.2 ± 12.9	63.9 ± 12.4	−8.10 ± 19.9	80.5 ± 20.6	75.1 ± 22.3	−5.69 ± 28.6	0.360	0.177	0.827
Lipids (g/day)	60.6 ± 22.6	56.4 ± 25.4	−6.32 ± 13.9	75.5 ± 27.8	78.52 ± 37.2	1.47 ± 30.7	0.096	0.091	0.654
Carbohydrates (g/day)	257 ± 56.2	242 ± 72.2	−14.8 ± 73.8	253 ± 72.4	264 ± 91.4	0.21 ± 100	0.879	0.888	0.809
TDF (g/day)	22.0 ± 6.67	23.1 ± 11.14	−1.47 ± 11.9	22.6 ± 11.9	22.5 ± 9.73	−2.41 ± 14.3	0.851	0.944	0.873

Table notes: Values are expressed as mean ± standard deviation. Δ = Week 12 − baseline. IDF: insoluble dietary fiber. SDF: soluble dietary fiber. TDF: total dietary fiber. Dietary fiber intake refers exclusively to dietary fiber obtained from food sources and does not include the study supplement (an additional 5 g/day). * *p*-value for between-group comparisons of baseline values (IDF vs. SDF) using the independent-samples Student’s *t*-test or the Mann–Whitney *U* test, as appropriate according to data distribution. ‡ *p*-value for between-group comparisons at Week 12 (IDF vs. SDF) using the independent-samples Student’s *t*-test or the Mann–Whitney *U* test, as appropriate according to data distribution. † *p*-value for between-group comparisons of changes from baseline (Δ = Week 12 − baseline) using the independent-samples Student’s *t*-test or the Mann–Whitney *U* test, as appropriate according to data distribution.

**Table 3:** Blood pressure at baseline, Week 12, and changes during the intervention according to treatment group.

Variable	IDF Group (n = 16)	SDF Group (n = 19)	Group Effect	Time Effect	Group × Time Interaction
**SBP (mmHg)**					
Baseline	148 ± 17.3	147 ± 19.1	F(1,34) = 1.132; *p* = 0.299	F(1,34) = 6.340; *p* = 0.020	F(1,34) = 0.018; *p* = 0.893
Week 12	136 ± 17.0	136 ± 20.9			
Change (Δ)	−11.6 ± 17.0	−11.2 ± 20.9			
**DBP (mmHg)**					
Baseline	82.7 ± 11.2	83.8 ± 13.8	F(1,34) = 0.870; *p* = 0.362	F(1,34) = 0.047; *p* = 0.830	F(1,34) = 0.718; *p* = 0.406
Week 12	81.8 ± 10.8	82.5 ± 13.2			
Change (Δ)	−0.9 ± 9.8	−1.3 ± 10.5			

Table notes: Data are presented as mean ± standard deviation (SD). Statistical analysis was performed using a two-way mixed repeated-measures ANOVA (group × time), with group as a between-subjects factor and time as a within-subjects factor; degrees of freedom were df(1,34) for all effects. The “Group Effect” column assesses differences between the groups (IDF vs. SDF) across all measurements; “Time Effect” assesses the overall change over time (baseline vs. Week 12); and “Group × Time Interaction” assesses whether the magnitude of change over time differs significantly between the two groups. Δ represents the absolute change from baseline to Week 12 (Week 12 value − baseline value). SBP = systolic blood pressure; DBP = diastolic blood pressure. IDF and SDF cor-respond to the two experimental groups as defined in the Methods section. Statistical significance was set at *p* < 0.05.

**Table 4:** Spearman’s rank correlation coefficients (ρ) or Pearson’s correlation coefficients (*r*) between changes in the relative abundance of gut bacterial taxa and changes in BP after the 12-week intervention.

	Systolic Blood Pressure (SBP)			Diastolic Blood Pressure (DBP)			
	IDF	SDF	TDF	IDF	SDF	TDF	
Total bacteria	−0.67	0.60	−0.03	−0.07	0.30	0.06	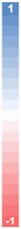
*Bacteroidetes*	−0.20	−0.70	−0.36	−0.64	0.00	−0.35	
*Firmicutes*	−0.60	0.40	−0.14	−0.50	0.90 **	−0.07	
*Verrucomicrobia*	−0.41	0.60	0.20	0.50	0.30	0.42	
*Akkermansia muciniphila*	−0.67	−0.70	−0.39	0.00	0.00	0.04	
*Bifidobacterium* spp.	−0.31	0.30	0.10	−0.21	0.90 *	0.19	
*Lactobacillus* spp.	−0.32	0.60	0.20	−0.63	0.30	−0.14	
Butyrate-producing bacteria	−0.25	−0.50	−0.15	0.04	−0.10	0.20	

IDF: insoluble dietary fiber; SDF: soluble dietary fiber; TDF: total dietary fiber; SBP: systolic blood pressure; DBP: diastolic blood pressure. Data are presented for the IDF, SDF, and TDF groups. * *p* < 0.05; ** *p* < 0.01.

**Table 5:** Spearman’s rank correlation coefficients (ρ) or Pearson’s correlation coefficients (*r*) between changes in serum biomarkers and changes in BP parameters after the 12-week intervention (n = 35).

A	SBP			DBP			
Biomarker	IDF	SDF	TDF	IDF	SDF	TDF	
TNF-α	−0.33	−0.25	−0.21	−0.33	0.21	0.04	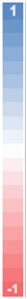
IL-6	−0.09	−0.23	−0.12	−0.24	0.11	−0.17	
IL-1b	−0.39	0.40	−0.19	−0.47	0.30	−0.21	
IL-8	−0.10	0.15	−0.09	−0.19	0.30	−0.11	
IL-10	0.02	−0.37	−0.12	0.10	0.02	0.07	
Cortisol	0.22	−0.58	−0.18	0.04	−0.32	−0.21	
FGF-23	0.30	0.03	0.05	−0.18	0.05	−0.24	
Leptin	0.10	−0.07	−0.01	0.35	−0.08	0.15	
Resistin	0.41	−0.13	0.05	0.04	−0.25	0.15	
PYY	0.13	0.11	0.07	−0.19	0.04	−0.20	
Ghrelin	0.35	0.03	0.25	0.23	−0.27	−0.03	
C-peptide	0.42	−0.05	0.09	−0.02	0.54	0.40	
GLP-1	−0.04	−0.51	−0.23	−0.01	0.12	0.17	
Zonulin	−0.08	−0.47	−0.25	−0.10	−0.06	−0.02	

IDF: insoluble dietary fiber; SDF: soluble dietary fiber; TDF: total dietary fiber; SBP: systolic blood pressure; DBP: diastolic blood pressure; IL: interleukin; TNF-α: tumor necrosis factor-α; FGF-23: fibroblast growth factor 23; GLP-1: glucagon-like peptide-1; PYY: peptide YY.

## Data Availability

The datasets generated and/or analyzed during the current study are available from the corre-sponding author upon reasonable request due to privacy and confidentiality restrictions, as the data contain potentially identifiable participant information and the informed consent agreements do not permit public data sharing.

## Abbreviations

The following abbreviations are used in this manuscript:

ANOVA	Analysis of variance
BMI	Body mass index
BP	Blood pressure
Cq	Quantification cycle
DBP	Diastolic blood pressure
GLP-1	Glucagon-like peptide-1
HR	Hazard ratio
IDF	Insoluble dietary fiber
IL	Interleukin
MCC	Microcrystalline cellulose
PCR	Polymerase chain reaction
PYY	Peptide YY
qPCR	Quantitative real-time polymerase chain reaction
SBP	Systolic blood pressure
SCFAs	Short-chain fatty acids
SDF	Soluble dietary fiber
SD	Standard deviation
T2D	Type 2 diabetes
TDF	Total dietary fiber
TNF-α	Tumor necrosis factor alpha
